# From Airway Inflammation to Molecular Remodeling: Integrating YKL-40, MBL and Epigenetic Biomarkers in Asthma and COPD

**DOI:** 10.3390/biom16081114

**Published:** 2026-07-30

**Authors:** Simona Maria Borta, Adrian Silviu Crișan, Romana Olivia Popețiu, Paula Alexandra Vulciu, Oana Știrbu, Cecilia Roberta Avram, Denisa Goldiș, Larisa Alexandra Rus, Darius Radu Roman, Alexandru Chioreanu, Radmila-Anca Bugari, Dana Zdremțan, Cristina Georgiana Firu, Imola Donath-Miklos

**Affiliations:** 1 Department of Internal Medicine, Faculty of Medicine, “Vasile Goldiș” Western University from Arad, B-dul Revoluției nr. 94-96, 310025 Arad, Romania; simoborta@gmail.com (S.M.B.); oanastirbu66@gmail.com (O.Ș.); rus.larisa@uvvg.ro (L.A.R.); 2 Department of Intensive Care and Emergency Medicine, Faculty of Medicine, “Vasile Goldiș” Western University from Arad, B-dul Revoluției nr. 94-96, 310025 Arad, Romania; crisan.adrian@uvvg.ro; 3 Department of Biochemistry, Faculty of Medicine, “Vasile Goldiș” Western University from Arad, B-dul Revoluției nr. 94-96, 310025 Arad, Romania; vulciu.paula@uvvg.ro (P.A.V.); zdremtan.dana@uvvg.ro (D.Z.); 4 Department of Biology and Life Science, Faculty of Medicine, “Vasile Goldiș” Western University from Arad, B-dul Revoluției nr. 94-96, 310025 Arad, Romania; avram.cecilia@uvvg.ro (C.R.A.); goldis.denisa@uvvg.ro (D.G.); 5 Clinical Laboratory of Medical Analyses, Arad County Emergency Clinical Hospital, Str. Andrényi Károly nr 2-4, 310037 Arad, Romania; 6 Doctoral School of Biomedical Sciences, Faculty of Medicine and Pharmacy, University of Oradea, Str. Universității nr 1, 410087 Oradea, Romania; roman.dariusradu@student.uoradea.ro; 7 Department of Otorinolaryngology, Faculty of Medicine, “Vasile Goldiș” Western University from Arad, B-dul Revoluției nr. 94-96, 310025 Arad, Romania; chioreanu.alexandru@uvvg.ro (A.C.); radmilabugariturcin@gmail.com (R.-A.B.); 8 Department of Hematology, Faculty of Medicine, “Vasile Goldiș” Western University from Arad, B-dul Revoluției nr. 94-96, 310025 Arad, Romania; firu.cristina@uvvg.ro

**Keywords:** asthma, chronic obstructive pulmonary disease, COPD, YKL-40, mannose-binding lectin, MBL, epigenetic remodeling, molecular phenotyping, precision medicine, biomarkers

## Abstract

Background: Asthma and chronic obstructive pulmonary disease (COPD) are heterogeneous chronic airway disorders characterized by complex interactions among inflammation, immune dysregulation, environmental exposures, and tissue remodeling. Conventional clinical classifications based on symptoms, lung function, and exacerbation history often fail to fully capture the biological mechanisms underlying disease progression and therapeutic variability. Methods: This narrative review summarizes current evidence regarding the biological and clinical significance of YKL-40, mannose-binding lectin (MBL), and epigenetic remodeling in asthma and COPD, with particular emphasis on the inflammation–epigenetic axis and its potential role in disease phenotyping and precision medicine. Results: Available evidence suggests that YKL-40, MBL, and epigenetic signatures represent complementary biomarker layers reflecting tissue remodeling, innate immune variability, and cumulative inflammatory adaptation, respectively. Chronic inflammatory signaling, oxidative stress, and epigenetic remodeling may contribute to persistent molecular memory and phenotypic stabilization, providing a mechanistic framework for understanding disease heterogeneity and progression. Advances in multi-omics technologies, artificial intelligence, and machine learning are further supporting the development of integrated multimarker models. Conclusions: YKL-40, MBL, and epigenetic signatures should currently be considered complementary research variables rather than validated clinical tools. Their translation requires disease-specific assay standardization, prospective evaluation against prespecified outcomes, external validation, and evidence that biomarker-guided decisions improve patient care.

## 1. Introduction

Asthma and chronic obstructive pulmonary disease (COPD) are among the most prevalent chronic respiratory disorders worldwide. They represent major causes of morbidity, healthcare utilization, and impaired quality of life. Although considerable progress has been made in understanding their pathophysiology, both diseases are highly heterogeneous, with substantial differences in inflammatory profiles, disease progression, treatment response, and long-term outcomes. Current diagnostic and monitoring strategies rely mainly on symptoms, pulmonary function testing, imaging, and a limited set of inflammatory markers. While these approaches are essential for diagnosis and disease monitoring, they provide only limited insight into the biological mechanisms underlying disease heterogeneity. This has stimulated growing interest in molecular biomarkers that may improve disease phenotyping, risk stratification, and the development of more personalized therapeutic strategies [1,2].

Chronic airway inflammation represents a central mechanism in both asthma and COPD. There is a complex interaction between innate and adaptive immunity, oxidative stress, epithelial injury, and extracellular matrix remodeling. These processes may damage the airway locally and may induce systemic biological changes that are reflected by circulating biomarkers [3,4,5,6,7,8,9,10,11]. Evidence suggests that repeated inflammatory and environmental injury leave persistent molecular imprints within airway epithelial and immune cells through transcriptional and epigenetic reprogramming. Therefore, chronic airway inflammation may promote long-lasting molecular remodeling that contributes to disease progression and phenotypic stability [12,13].

Although asthma and COPD share several inflammatory and remodeling mechanisms, they differ substantially in their predominant inflammatory endotypes. Asthma is classically characterized by type 2 eosinophilic inflammation, whereas COPD is more frequently associated with neutrophilic and macrophage-dominated inflammatory responses, frequently involving Th1- and Th17-related pathways. The biological relevance and clinical interpretation of individual biomarkers may differ between diseases and even across endotypes within the same disease [1,2,14,15,16,17].

Among emerging inflammatory biomarkers, YKL-40 (chitinase-3-like protein 1, CHI3L1) has attracted considerable interest due to its involvement in macrophage activation, tissue remodeling, angiogenesis, and chronic inflammatory signaling. Elevated circulating CHI3L1/YKL-40 concentrations have been reported in patients with asthma and COPD, where they have been associated with disease severity, airflow limitation, inflammatory phenotypes, and acute exacerbations. Recent studies suggest that CHI3L1/YKL-40 may function as a marker of inflammation and an active participant in airway remodeling and disease progression, although the relative contribution of these mechanisms remains unclear [9,10,11,18,19].

Another important component of innate immunity is mannose-binding lectin (MBL). MBL is a pattern-recognition molecule that initiates the lectin pathway of complement activation. Genetic polymorphisms within the MBL2 gene can influence circulating MBL levels and functional activity, contributing to inter-individual variability in immune responses. Altered MBL expression and MBL2 variants may influence susceptibility to allergic inflammation, asthma phenotypes, respiratory infections, and dysregulated inflammatory responses. These observations suggest that MBL may reflect inherited innate immune variability while also influencing disease progression through its effects on pathogen recognition and clearance [20,21,22,23,24,25,26,27].

Beyond inflammatory proteins, epigenetic mechanisms appear as important regulators of chronic respiratory disease pathogenesis. DNA methylation, histone modifications, and non-coding RNAs integrate genetic susceptibility with environmental exposures such as cigarette smoke, air pollution, allergens, and respiratory infections. These molecular alterations contribute to persistent inflammatory activation, airway remodeling, and disease progression. Recent epigenome-wide studies have identified that DNA methylation signatures may be indicators for asthma severity, airflow limitation, and COPD-related inflammatory pathways, supporting their potential role as biomarkers of long-term biological remodeling [28,29,30,31,32,33,34].

Rather than representing independent biomarkers, CHI3L1/YKL-40, MBL, and epigenetic modifications may be interpreted as interconnected biological layers within the same disease continuum. Together, these layers provide a framework for understanding how airway inflammation may evolve from a dynamic immune response into a stabilized disease phenotype characterized by persistent remodeling, altered inflammatory memory, and variable therapeutic responsiveness [10,13,25,33,35].

The present review examines asthma and COPD through the integrated perspective of inflammation-driven molecular remodeling. Emphasis is placed on the interaction between innate immune variability, remodeling-associated inflammatory mediators, and epigenetic regulation, with CHI3L1/YKL-40, mannose-binding lectin, and epigenetic signatures serving as representative biomarkers of these interconnected processes. By framing chronic airway inflammation as a systems biology phenomenon with potential molecular memory, this review discusses the relevance of multimarker models for disease phenotyping, risk stratification, and future precision medicine strategies in chronic respiratory disease.

## 2. Material and Methods

### 2.1. Literature Search and Review Scope

This narrative review was designed to provide an integrated overview of the current evidence regarding CHI3L1/YKL-40, mannose-binding lectin (MBL), and epigenetic remodeling in asthma and chronic obstructive pulmonary disease (COPD), with particular emphasis on their biological roles, clinical relevance, and potential integration into precision medicine frameworks.

A structured literature search was conducted using PubMed/MEDLINE, Scopus, Web of Science, and Google Scholar databases. The search strategy combined relevant keywords and Medical Subject Headings (MeSH) terms, including “asthma”, “COPD”, “YKL-40”, “CHI3L1”, “mannose-binding lectin”, “MBL”, “MBL2 polymorphism”, “epigenetics”, “DNA methylation”, “DNA hydroxymethylation”, “histone modification”, “microRNA”, “airway remodeling”, “biomarkers”, and “precision medicine”. Additional studies were identified through manual screening of reference lists from selected articles.

Priority was given to original research articles, systematic reviews, meta-analyses, and consensus documents published between 2020 and 2025. Earlier landmark studies were included when considered essential for understanding the biological mechanisms or historical development of the field. Articles were selected based on their scientific relevance, methodological quality, translational value, and contribution to the understanding of inflammatory and epigenetic pathways involved in chronic airway diseases. Both positive and negative findings were considered during the narrative synthesis, with particular attention to areas of inconsistency, conflicting evidence, and unresolved questions regarding biomarker utility in asthma and COPD.

Given the narrative nature of this review, formal systematic review procedures, study quality scoring, and quantitative meta-analytic methods were not applied. Instead, the available evidence was critically synthesized to highlight emerging mechanistic concepts, biomarker interactions, and future research directions relevant to personalized respiratory medicine.

### 2.2. Use of Generative Artificial Intelligence

Generative artificial intelligence (GenAI) tools, including ChatGPT (OpenAI GPT-5.5 series, San Francisco, CA, USA), were used to assist with language editing, manuscript structuring, conceptual organization, drafting support, figure conceptualization, and refinement of scientific text.

All references, scientific statements, interpretations, tables, figures, and conclusions were independently reviewed, verified, revised, and approved by the authors.

The authors assume full responsibility for the accuracy, integrity, originality, and scientific validity of all content presented in this manuscript.

## 3. Results: Chronic Airway Inflammation as a Systems Biology Process

Chronic airway diseases are increasingly recognized as complex systems-level disorders involving dynamic interactions among immune cells, structural airway components, environmental exposures, genetic predisposition, and epigenetic regulation. These mechanisms collectively contribute to disease heterogeneity, progression, and variable therapeutic responses, supporting the concept of chronic airway inflammation as a systems biology process rather than a single-pathway disorder [4,13,14,15,16,33].

### 3.1. Innate Immunity

Innate immunity constitutes the first line of defense against inhaled pathogens, allergens, environmental pollutants, and tissue injury. Airway epithelial cells, resident macrophages, dendritic cells, and recruited neutrophils form a highly coordinated surveillance system capable of detecting danger-associated molecular patterns (DAMPs) and pathogen-associated molecular patterns (PAMPs) through pattern recognition receptors (PRRs), including Toll-like receptors (TLRs) and NOD-like receptors (NLRs). Activation of these pathways induces the production of pro-inflammatory cytokines, chemokines, and growth factors that initiate and sustain chronic airway inflammation [15,16,17].

#### 3.1.1. Macrophages

Macrophages represent one of the most abundant immune cell populations within the respiratory tract and play a central role in maintaining pulmonary homeostasis. In healthy airways, alveolar macrophages contribute to pathogen clearance, tissue repair, and resolution of inflammation. However, chronic exposure to cigarette smoke, allergens, airborne pollutants, and recurrent infections promotes macrophage activation and phenotypic reprogramming. Activated macrophages release multiple inflammatory mediators, including tumor necrosis factor-alpha (TNF-α), interleukin-1 beta (IL-1β), interleukin-6 (IL-6), transforming growth factor-beta (TGF-β), and reactive oxygen species (ROS), thereby contributing to chronic inflammation and tissue injury [4,15,36,37,38].

Macrophage polarization has emerged as an important determinant of disease phenotype. Classically activated M1 macrophages predominantly promote inflammatory responses through cytokine production and oxidative stress, whereas alternatively activated M2 macrophages contribute to tissue remodeling, fibrosis, extracellular matrix deposition. Increasing evidence suggests that persistent imbalance between M1 and M2 phenotypes contributes to both asthma severity and COPD progression, linking inflammatory activity with long-term tissue remodeling [15,36,38,39,40,41,42].

#### 3.1.2. Neutrophils

Neutrophilic inflammation is increasingly recognized as a major contributor to severe asthma and advanced COPD [1,2]. Activated neutrophils release proteolytic enzymes, including neutrophil elastase, matrix metalloproteinases (MMPs), and myeloperoxidase, which directly damage epithelial barriers and extracellular matrix structures. Excessive neutrophil recruitment is associated with accelerated lung function decline, mucus hypersecretion, increased exacerbation frequency, and corticosteroid resistance [43,44,45].

Beyond their antimicrobial functions, neutrophils participate in chronic inflammatory signaling through the generation of neutrophil extracellular traps (NETs), ROS production, and activation of NF-κB-dependent pathways. These mechanisms amplify local inflammation and create a self-sustaining inflammatory microenvironment that promotes progressive airway injury [45,46,47,48].

#### 3.1.3. Dendritic Cells

Dendritic cells serve as key mediators linking innate and adaptive immunity. Located within airway mucosal tissues, they continuously sample inhaled antigens and environmental stimuli. Upon activation, dendritic cells migrate to regional lymph nodes, where they present processed antigens to naïve T lymphocytes and initiate adaptive immune responses [49,50,51].

In asthma, dendritic cells play a critical role in promoting Th2 polarization and allergic sensitization [16,17], whereas in COPD they contribute to chronic inflammatory activation through interactions with Th1 and Th17 pathways [42,51,52]. Recent evidence also indicates that dendritic cells contribute to epithelial remodeling and shape long-term immune responses, thereby influencing the chronic airway inflammation [16,17,50,51,52,53].

### 3.2. Adaptive Immunity

Although innate immune activation initiates airway inflammation, adaptive immune responses largely determine disease phenotype, chronicity, and progression. The balance among T-helper cell subsets profoundly influences cytokine profiles, inflammatory pathways, and structural airway alterations. Distinct adaptive immune signatures have been associated with different asthma endotypes and COPD phenotypes, highlighting their importance in disease heterogeneity [14,15,16,42,52,54,55].

#### 3.2.1. Th1 Responses

Th1 lymphocytes produce interferon-gamma (IFN-γ), TNF-α, and other pro-inflammatory mediators that enhance macrophage activation and cellular immunity [3,16,52,56]. Th1-dominant inflammation is particularly prominent in COPD and is associated with chronic tissue injury and remodeling [4,15,56].

#### 3.2.2. Th2 Responses

Th2-driven inflammation represents the classical immunological hallmark of allergic asthma. Cytokines such as IL-4, IL-5, and IL-13 promote eosinophilic recruitment, IgE production, mucus hypersecretion, and airway hyperresponsiveness. Advances in molecular phenotyping have demonstrated that T2-high/T2-low asthma represent biologically distinct inflammatory patterns with different therapeutic responses [53,57,58].

#### 3.2.3. Th17 Responses

Th17 cells have emerged as important contributors to chronic airway inflammation through the production of IL-17A, IL-17F, and related cytokines [59]. These mediators stimulate neutrophil recruitment, epithelial activation, and sustained inflammatory signaling. Increased Th17 activity has been associated with severe asthma, neutrophilic phenotypes, COPD progression, and treatment resistance [52]. The simultaneous activation of Th1, Th2, and Th17 pathways may explain the substantial biological heterogeneity observed in chronic airway diseases [16,17,52,55,56,59].

### 3.3. Tissue Remodeling

Tissue remodeling represents a hallmark of chronic respiratory disease and reflects the cumulative effects of cytokine signaling, immune cell infiltration, oxidative stress, and dysregulated repair mechanisms. Remodeling processes may become partially independent of active inflammation, contributing to irreversible airflow limitation and disease progression [7,8,60,61,62].

#### 3.3.1. Fibrosis

Fibrotic remodeling is primarily driven by chronic activation of fibroblasts and myofibroblasts in response to TGF-β, IL-13, and other profibrotic mediators. Extracellular matrix accumulation contributes to airway wall thickening and reduced tissue elasticity. Fibrosis has been documented in both severe asthma and COPD, where it is associated with impaired pulmonary function and long-term structural damage [61,63,64,65].

#### 3.3.2. Extracellular Matrix Remodeling

The extracellular matrix (ECM) is increasingly recognized as an active regulator of airway biology rather than a passive structural scaffold. Chronic inflammation disrupts the balance between matrix synthesis and degradation, resulting in abnormal collagen turnover, elastin fragmentation, altered proteoglycan composition, and matrix stiffness [63]. Matrix metalloproteinases released by macrophages and neutrophils play a central role in this process and contribute to progressive tissue destruction [4,8,42,66,67,68].

#### 3.3.3. Airway Remodeling

Airway remodeling encompasses epithelial injury, goblet cell hyperplasia, subepithelial fibrosis, smooth muscle hypertrophy, angiogenesis, and alterations in extracellular matrix architecture. These structural changes contribute directly to airflow limitation, airway hyperresponsiveness, mucus production, and disease progression [7,8,61,62,65]. Importantly, biomarkers such as CHI3L1/YKL-40 have been linked to remodeling-associated pathways, supporting their potential utility in clinical activity [10,69,70,71,72,73].

**Figure 1:**
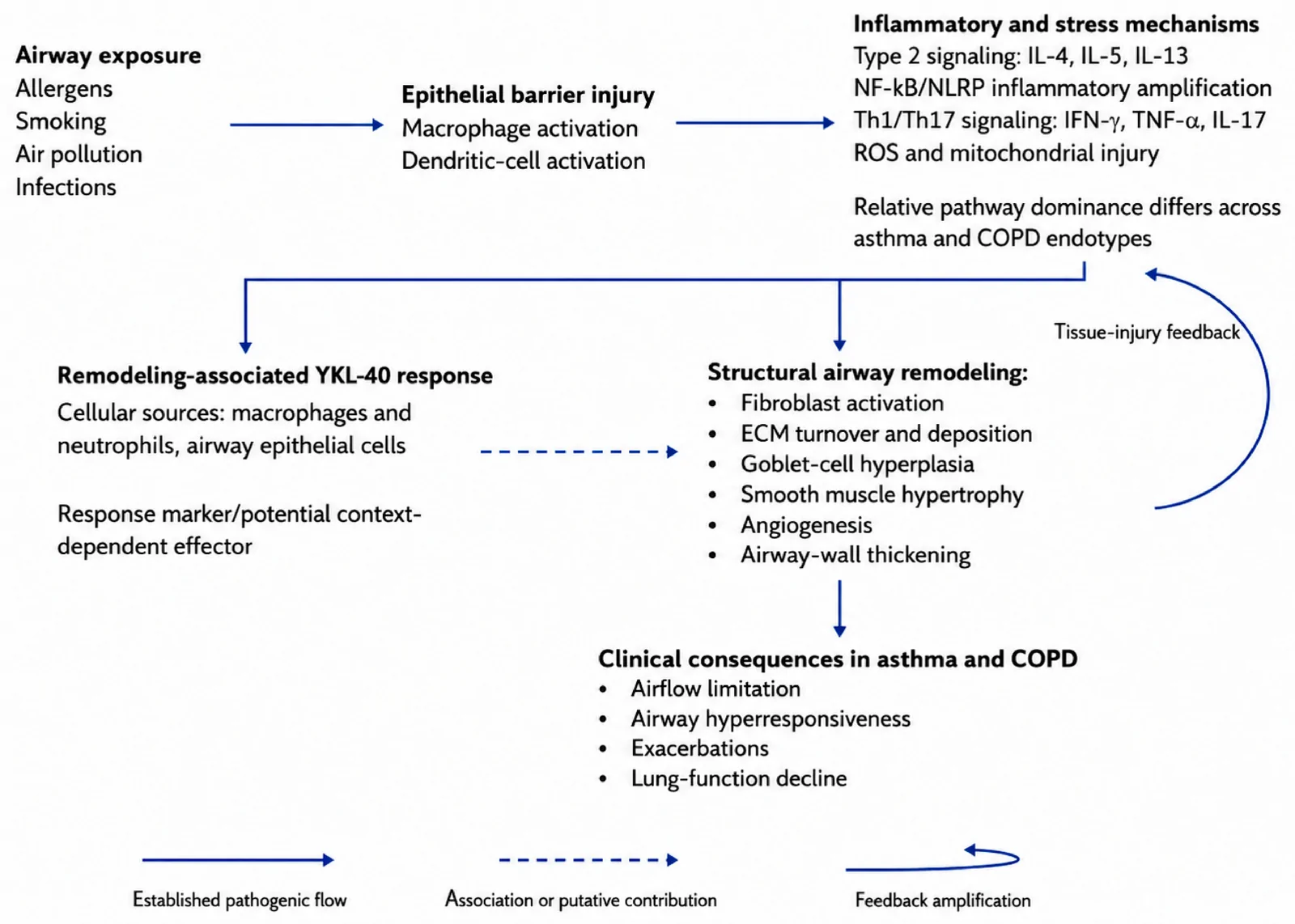
Systems-level pathway linking airway exposure, inflammatory activation, and structural remodeling in asthma and COPD. Allergens, tobacco smoke, air pollution, and respiratory infections induce epithelial injury and activate innate and adaptive immune responses [1,2,14,15,16,28]. Cytokine signaling and oxidative stress promote fibroblast activation, extracellular-matrix turnover, mucus-cell hyperplasia, angiogenesis, and airway smooth-muscle remodeling [4,6,8,48,62,63,73]. CHI3L1/YKL-40 is positioned as a remodeling-associated response marker and potential context-dependent effector rather than an obligatory intermediate [10,18,71,73,74,75]. Persistent remodeling and tissue injury can further amplify inflammatory signaling, contributing to airflow limitation, may increase the risk of exacerbations, and lung-function decline. Solid arrows indicate pathogenic progression, whereas dashed arrows indicate association or a putative context-dependent contribution [7,8,48,61,62].

The pathophysiological relationships among environmental exposures, inflammatory activation, tissue remodeling, and CHI3L1/YKL-40 expression are summarized in Figure 1.

## 4. CHI3L1/YKL-40 as a Remodeling-Associated Biomarker

### 4.1. Molecular Biology of CHI3L1/YKL-40

YKL-40, also known as chitinase-3-like protein 1 (CHI3L1), is a highly conserved glycoprotein belonging to the family of mammalian chitinase-like proteins. It is encoded by the CHI3L1 gene located on chromosome 1q32.1. Unlike true chitinases, CHI3L1/YKL-40 lacks enzymatic chitin-degrading activity due to mutations within its catalytic domain. Nevertheless, it retains the ability to bind chitin, heparin, collagen, and extracellular matrix (ECM) components, suggesting important regulatory functions in tissue remodeling, inflammation, and cellular signaling [10,74,75].

Its expression is induced by cytokine activation, oxidative stress, tissue injury, and extracellular matrix turnover, placing CHI3L1/YKL-40 at the interface between innate immune activation and structural airway adaptation [10,74,75].

Available mechanistic evidence does not support a single universal CHI3L1/YKL-40 pathway. Subsequent evidence has continued to implicate CHI3L1/YKL-40 in fibroblast proliferation, myofibroblast differentiation, and extracellular matrix accumulation, although direct independent replication of these specific signaling effects remains limited [71,76,77].

In human bronchial smooth-muscle cells, CHI3L1/YKL-40 promotes proliferation and migration through protease-activated receptor 2 (PAR-2)-dependent signaling [73]. In separate epithelial and macrophage models, CHI3L1/YKL-40 binding to IL-13Rα2 recruits TMEM219 for ERK and AKT signaling, whereas Wnt/β-catenin activation is IL-13Rα2-dependent but TMEM219-independent [78]. These findings indicate that the effects of CHI3L1/YKL-40 are cell- and context-dependent.

The same biological breadth limits disease specificity. CHI3L1/YKL-40 is elevated in multiple inflammatory, fibrotic, cardiovascular, and malignant conditions [10,74,75]. A circulating CHI3L1/YKL-40 concentration therefore cannot identify asthma, COPD, or a specific airway endotype in isolation. Its respiratory interpretation should be conditional on the clinical diagnosis, sampling compartment, exacerbation status, smoking exposure, and complementary measures of airway inflammation and remodeling [18,68,69,71,79].

Although increasing evidence supports the role of CHI3L1/YKL-40 in airway remodeling, current clinical studies do not fully distinguish its contribution to acute inflammatory activity from its association with chronic structural remodeling. Most available evidence is derived from cross-sectional studies and circulating CHI3L1/YKL-40 concentrations likely reflect both biological processes simultaneously. Longitudinal studies integrating biomarker measurements with imaging, lung function, and histopathological assessment are needed to better define the remodeling-specific value of CHI3L1/YKL-40 [18,71].

### 4.2. CHI3L1/YKL-40 in Asthma

The role of CHI3L1/YKL-40 in asthma has attracted increasing attention over the past decade due to its association with disease severity, airway remodeling, and inflammatory heterogeneity. Elevated serum and sputum CHI3L1/YKL-40 concentrations have consistently been reported in patients with asthma compared with healthy controls, supporting its potential utility as a biomarker of disease activity. Meta-analytic evidence suggests that CHI3L1/YKL-40 levels correlate with asthma severity and may contribute to the differentiation of clinical phenotypes [19,69,80].

Mechanistically, CHI3L1/YKL-40 appears to contribute to several pathological processes relevant to asthma. Experimental studies have demonstrated its involvement in allergen sensitization, eosinophilic recruitment, immunoglobulin E (IgE) production [81], and airway smooth muscle proliferation [73]. Moreover, increased CHI3L1/YKL-40 expression has been associated with subepithelial membrane thickening and airway remodeling [72,82].

Importantly, CHI3L1/YKL-40 may also reflect inflammatory endotype diversity. While asthma has traditionally been regarded as a Th2-driven eosinophilic disorder, increasing evidence indicates that CHI3L1/YKL-40 is particularly associated with neutrophilic and mixed inflammatory phenotypes [18,19,69,72,83]. Elevated CHI3L1/YKL-40 concentrations have been linked to poor symptom control, reduced lung function and therapy resistance, supporting its role as a biomarker of complex inflammatory remodeling rather than isolated eosinophilic inflammation [18,19,69,83].

Clinical studies, including data from Caucasian asthma and COPD cohorts, support the potential value of serum CHI3L1/YKL-40 for distinguishing inflammatory airway phenotypes and identifying patients with higher inflammatory burden. In advanced COPD, elevated CHI3L1/YKL-40 has also been associated with exacerbation status and systemic leukocyte profiles, suggesting that it may provide complementary information beyond conventional functional assessment [9,11].

### 4.3. CHI3L1/YKL-40 in COPD

Circulating CHI3L1/YKL-40 concentrations are generally higher in patients with COPD than in healthy controls and have shown inverse cross-sectional associations with pulmonary function [9,11,71,79,84]. However, substantial between-study heterogeneity, limited disease specificity, and the absence of standardized clinical thresholds restrict its diagnostic interpretation [10,18,84].

Mechanistic observations support this interpretation. Alveolar macrophages represent an important pulmonary source of YKL-40, and experimental exposure to CHI3L1/YKL-40 increases the release of IL-8, MCP-1, MIP-1α, and MMP-9, linking macrophage activation to inflammatory and proteolytic signaling [68].

In primary human lung fibroblasts, CHI3L1/YKL-40 has been shown to promote proliferation, migration, α-SMA expression, and collagen I/III production, with collagen induction involving ERK and p38 MAPK signaling [71,77].

These mechanisms are compatible with remodeling-associated activity, but they do not establish that circulating CHI3L1/YKL-40 directly measures irreversible structural damage or predicts COPD progression [18,68,71,84].

### 4.4. Association with Exacerbations

Acute exacerbations are major clinical events in both asthma and COPD, contributing to accelerated disease progression, impaired quality of life, increased healthcare utilization, and, particularly in COPD, increased mortality. In both diseases, identifying patients with high risk of future exacerbations remains an important challenge, stimulating the search for biomarkers reflecting disease instability and inflammatory activity [1,2,14].

Several studies have reported higher CHI3L1/YKL-40 concentrations during disease instability or associations with exacerbation-related clinical phenotypes [9,11,50,71,80].

In the exploratory retrospective cross-sectional study conducted by our group, all patients with severe or very severe COPD were sampled during an acute exacerbation. Serum CHI3L1/YKL-40 strata were associated with distinct leukocyte profiles, including differences in neutrophil, lymphocyte, eosinophil, and basophil-related measures. However, CHI3L1/YKL-40 did not distinguish severe from very severe COPD, and stable-phase samples from the same patients were not available [9,11]. The study therefore supports an association between CHI3L1/YKL-40 and leukocyte profile during acute exacerbation of COPD, but it does not demonstrate prediction of future exacerbations or disease progression.

**Figure 2:**
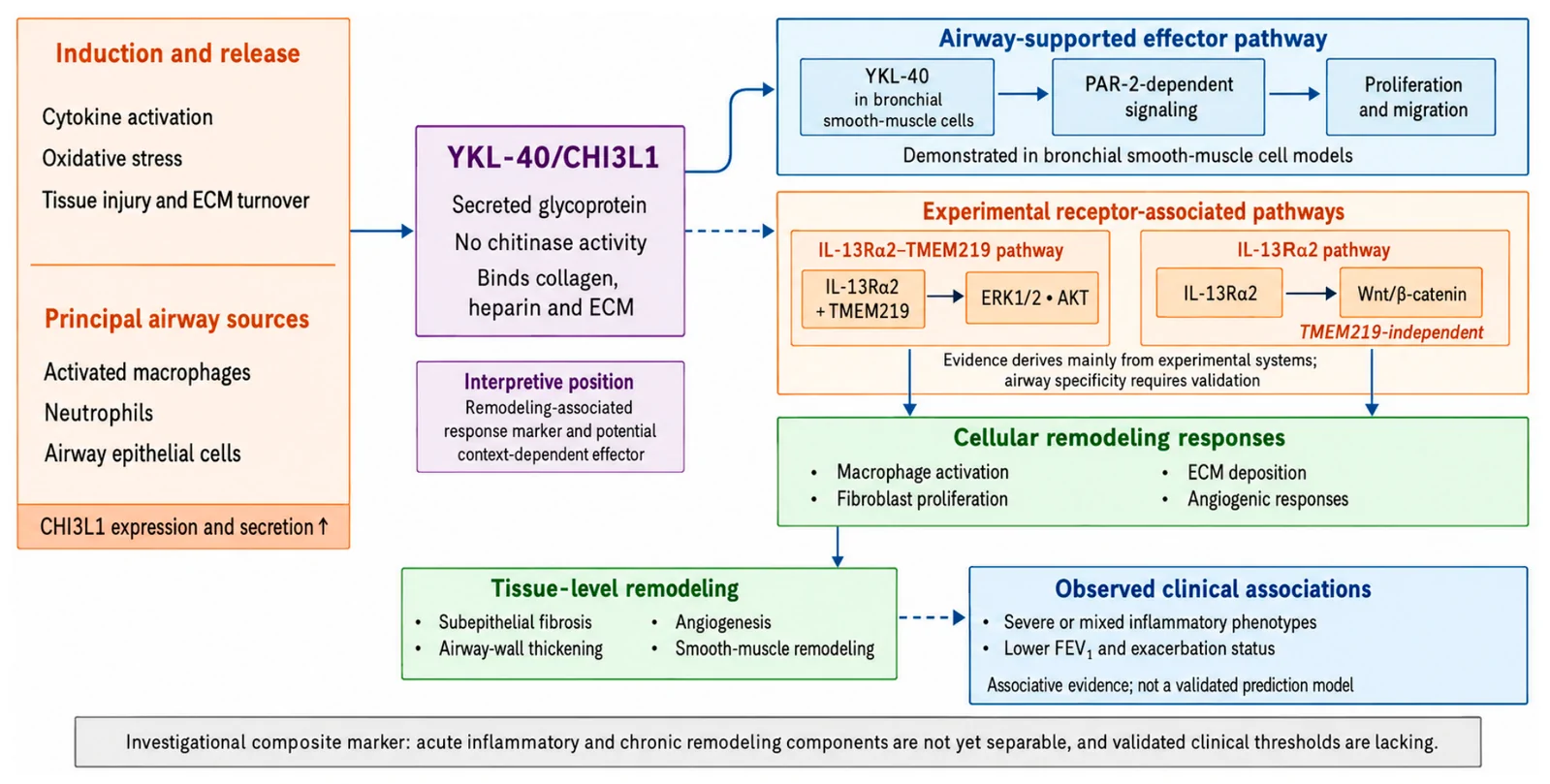
Mechanistic interpretation of CHI3L1/YKL-40 as a remodeling-associated response marker in asthma and COPD. In inflammatory and tissue-injury contexts, CHI3L1/YKL-40 is released by activated macrophages, neutrophils, and airway epithelial cells [68,69,74,75]. In human bronchial smooth-muscle cells, CHI3L1/YKL-40 promotes proliferation and migration through protease-activated receptor 2 (PAR-2)-dependent signaling [73]. In cellular models, CHI3L1/YKL-40 binding to IL-13Rα2 activates MAPK/ERK, AKT, and Wnt/β-catenin signaling, with TMEM219 participating in the receptor complex [78]. These experimentally defined, cell-specific pathways may contribute to macrophage activation, fibroblast and smooth-muscle responses, extracellular-matrix turnover, and structural remodeling, but they do not establish a single universal mechanism in human asthma or COPD [68,71,73,74,78]. Circulating CHI3L1/YKL-40 should therefore be interpreted as an investigational composite marker that does not yet distinguish acute inflammatory activity from chronic structural remodeling [11,18,71,79,84]. Solid arrows indicate direct experimental or biological support, whereas dashed arrows indicate context-dependent or associative links [68,73,78]. Box colors distinguish the main conceptual domains of the figure: orange, induction/release and experimental receptor-associated pathways; purple, CHI3L1/YKL-40 core interpretation; blue, airway-supported effector pathways and observed clinical associations; green, cellular and tissue-level remodeling responses; and grey, overall interpretive caution.

Prospective longitudinal cohorts with measurements obtained during both stable and exacerbation periods are required to determine whether serial CHI3L1/YKL-40 assessment improves prediction beyond established clinical variables such as previous exacerbations, symptoms, and pulmonary function [1,2,18,85]. Until such incremental and externally validated predictive value is demonstrated, CHI3L1/YKL-40 should be evaluated as a candidate variable within multimarker models rather than as a validated prognostic marker [18,42,84,85,86,87] (Figure 2).

## 5. Mannose-Binding Lectin and Innate Immune Variability

### 5.1. Lectin Pathway

Mannose-binding lectin (MBL) is a soluble pattern-recognition molecule that plays a central role in innate immune defense. Produced primarily by hepatocytes, MBL recognizes conserved carbohydrate structures expressed on the surfaces of bacteria, viruses, fungi, and other pathogens. Upon ligand binding, MBL associates with mannose-binding lectin-associated serine proteases (MASPs), initiating the lectin pathway of complement activation. This process results in opsonization, enhanced phagocytosis, inflammatory signaling, and pathogen elimination [25,88,89].

Unlike the classical complement pathway, which can be influenced by antigen–antibody interactions, the lectin pathway provides a rapid antibody-independent mechanism of host defense. Through activation of complement components C4, C2, and C3, MBL contributes to the early containment of infection and modulation of inflammatory responses. Accumulating evidence suggests that MBL may influence tissue injury and inflammatory homeostasis, supporting its role as a broader regulator of innate immune function rather than a simple antimicrobial molecule [25,88,89].

### 5.2. MBL2 Polymorphisms

The biological activity of MBL is largely determined by genetic variation within the MBL2 gene located on chromosome 10q21. Structural polymorphisms in exon 1 and regulatory polymorphisms within promoter regions substantially influence circulating MBL concentrations and functional activity. Three exon 1 variants, commonly designated B (rs1800450), C (rs1800451), and D (rs5030737), disrupt oligomer formation and reduce complement activation efficiency. These variants are frequently grouped as the O allele, whereas the wild-type allele is designated A [22,23,88,90].

Promoter polymorphisms, particularly the H/L and X/Y variants, further modulate gene expression and serum protein concentrations. The combined effect of structural and promoter variants generates considerable inter-individual variability in MBL production, ranging from high-producing haplotypes to profound functional deficiency [25,88,90].

### 5.3. Asthma Susceptibility

The relationship between MBL2 polymorphisms and asthma susceptibility remains complex and occasionally controversial. Because MBL participates in complement activation, pathogen clearance, and regulation of inflammatory responses, altered MBL function has been proposed as a potential contributor to asthma development [20,21,22,23,24]. Several studies have suggested that low-producing MBL2 variants may influence susceptibility to respiratory infections during childhood [26,27,89].

However, genetic association studies have produced heterogeneous and sometimes conflicting results across different ethnic populations. While several investigations suggested that low-producing MBL2 variants may influence susceptibility to specific asthma phenotypes, other well-designed case–control studies found no significant associations between MBL2 polymorphisms and asthma susceptibility, disease severity, pulmonary function, or serum IgE levels. Furthermore, a meta-analysis including nine studies concluded that the most frequently investigated MBL2 polymorphisms (codon 54 A/B, −550 H/L, and −221 X/Y) were not significantly associated with asthma risk. These discrepancies likely reflect differences in ethnicity, asthma endotypes, environmental exposures, study design, and sample size. Therefore, the current evidence suggests that MBL is more appropriately regarded as a potential modifier of disease phenotype than as a universal determinant of asthma susceptibility or severity [22,23,91,92].

### 5.4. Clinical Phenotypes

Beyond disease susceptibility, MBL may contribute to the clinical heterogeneity observed among patients with chronic airway disease. Variability in complement activation capacity can influence microbial clearance, inflammatory burden, frequency of respiratory infections, and exacerbation risk. Studies investigating respiratory disorders have reported associations between MBL deficiency, recurrent respiratory tract infections [26,89], and altered airway microbiota composition [93,94].

Genetically determined MBL deficiency may influence airway host–microbiota interactions, but its clinical implications in COPD remain uncertain. Dicker et al. associated MBL deficiency with greater airway microbiota diversity and a lower frequency of exacerbations, whereas Albert et al. found no association with the time to first exacerbation, exacerbation frequency, or exacerbation-related hospitalization. These findings do not support MBL deficiency as a general risk factor for COPD exacerbations. Instead, MBL2 genotype may represent a context-dependent modifier of airway microbial ecology and innate immune responsiveness, whose clinical effects require further investigation [93,94].

### 5.5. Personalized Inflammatory Phenotypes

From a precision medicine perspective, MBL2 genotype captures a stable inherited component of innate immune variability. Unlike dynamic inflammatory markers that fluctuate with disease activity, MBL concentrations are strongly influenced by inherited MBL2 haplotypes and the current inflammatory context. Therefore MBL reflects long-term differences in host immune responsiveness [25,88,90].

Within the framework proposed in this review, MBL can be viewed as one component of a multilayered inflammatory phenotype [21,22,25,88]. Whereas CHI3L1/YKL-40 reflects active remodeling and inflammatory tissue adaptation [10,18], MBL reflects inherited variability in pathogen recognition and complement activation. Integration of MBL-related information with remodeling-associated biomarkers and epigenetic signatures may improve disease stratification and facilitate the identification of clinically meaningful endotypes [30,32,33,86,87].

## 6. Epigenetic Remodeling in Chronic Airway Disease

Epigenetic remodeling provides a mechanistic link between environmental exposure, chronic inflammation, and persistent disease phenotypes in asthma and COPD. Unlike genetic variation, epigenetic regulation is dynamic and potentially reversible, yet it can generate stable transcriptional programs. In chronic airway disease, DNA methylation, DNA hydroxymethylation, histone modifications, and non-coding RNAs may therefore function not only as biomarkers, but also as molecular mediators of inflammatory memory and long-term airway adaptation [12,13,30,32,33].

The principal epigenetic mechanisms which can influence inflammatory memory, airway remodeling, and disease progression in asthma and COPD are summarized in Table 1.

### 6.1. DNA Methylation

DNA methylation is one of the most extensively studied epigenetic mechanisms in chronic respiratory disease. It involves the addition of methyl groups to cytosine residues, most commonly at CpG sites, and may influence gene expression by altering chromatin accessibility and transcription factor binding. In asthma and COPD, differential DNA methylation has been associated with immune signaling, epithelial dysfunction, corticosteroid responsiveness, airflow limitation, and disease severity [30,32,33,95].

Recent epigenome-wide studies have strengthened the concept that DNA methylation signatures may capture clinically relevant dimensions of airway disease biology. In asthma, methylation patterns have been linked to clinical markers, inflammatory traits, and disease heterogeneity, suggesting potential value for molecular phenotyping. In COPD, systematic analyses of epigenome-wide association studies indicate that methylation changes reflect cumulative gene–environment interactions across the life course, particularly those related to smoking exposure and lung function decline [30,32].

Within the framework of this review, DNA methylation should be interpreted as a molecular interface between exposure history and disease phenotype. Rather than serving as a single diagnostic marker, methylation profiles may help identify patients in whom chronic inflammatory activation has become biologically embedded [30,32,33,95].

### 6.2. DNA Hydroxymethylation

DNA hydroxymethylation, primarily involving the formation of 5-hydroxymethylcytosine (5hmC), represents an additional layer of epigenetic regulation. Although it has been less extensively investigated than DNA methylation in chronic airway disease, hydroxymethylation is increasingly recognized as a dynamic mark associated with active gene regulation, cellular differentiation, oxidative stress responses, and tissue-specific transcriptional plasticity [31,33,98].

5hmC is produced when TET1–3 oxidize 5mC and can function either as a relatively stable regulatory mark or as an intermediate in active DNA demethylation [33,98]. In asthma-relevant airway models, allergen-responsive TET1 signaling has been linked to epithelial interferon and aryl hydrocarbon receptor programs, suggesting that altered 5mC/5hmC turnover may influence allergic airway inflammation [31,33,105]. In human bronchial epithelial cells, exposure to diesel exhaust particles or house dust mite allergen increased TET1 and DNMT1 expression and induced genome-wide changes in 5mC and 5hmC, supporting exposure-responsive regulation of the airway methylome and hydroxymethylome [106].

However, many respiratory epigenome-wide studies rely on bisulfite-based methods that do not distinguish 5mC from 5hmC [32,98]. No replicated and externally validated 5hmC signature is currently available for assessing asthma severity, COPD-related airflow limitation, exacerbation risk, or treatment response; 5hmC should therefore be considered mechanistically informative but not clinically validated [29,31,32,33,98].

### 6.3. Histone Modifications

Histone modifications can alter inflammatory transcription by changing chromatin accessibility [33].

In a small pilot study of bronchial epithelial cells from four adults with asthma and three healthy controls, H3K27ac profiling identified 4321 differentially enriched regions, supporting asthma-associated alteration of the epithelial histone landscape, including the T2-associated POSTN, SERPINB2, CLCA1, and TSLP loci [29,100]. Because this was a small pilot study, the finding is relevant to endotype discovery but does not represent a validated biomarker.

Histone regulation may also contribute to treatment-refractory disease. Ligand-activated glucocorticoid receptors recruit HDAC2 to NF-κB-regulated inflammatory gene complexes, where HDAC2-mediated deacetylation contributes to transcriptional repression [107,108]. In smoking-related COPD, oxidative and nitrosative stress can reduce HDAC2 activity, weakening this repression and contributing to corticosteroid resistance [34,97,99,109,110]. Reduced HDAC2 activity has also been reported in bronchial biopsies and alveolar macrophages from patients with mild asthma [96,110]. These findings connect chromatin regulation to persistent inflammatory phenotypes, although histone marks are not yet validated for routine clinical stratification.

### 6.4. microRNA

MicroRNAs are small non-coding RNAs that regulate gene expression post-transcriptionally by promoting mRNA destabilization or translational repression. In asthma and COPD, altered microRNA expression has been implicated in immune regulation, epithelial function, mucus hypersecretion, oxidative stress, apoptosis, extracellular matrix remodeling, and corticosteroid response. Because individual microRNAs may regulate multiple target genes, they are particularly suited to modulating complex inflammatory networks rather than single linear pathways [101,102,103].

Recent studies support the potential role of microRNAs as biomarkers of disease phenotype and molecular activity in chronic airway disease. For example, Chang et al. reported increased miR-125b-5p expression in asthma–COPD overlap and linked it experimentally to oxidative stress and late apoptosis through IL6R/TRIAP1 signaling [101].

Within the conceptual framework of this review, microRNA profiles may provide information complementary to CHI3L1/YKL-40 and MBL-related variables.

However, microRNA research in asthma and COPD remains heterogeneous, with variability related to sample type, disease stage, treatment exposure, and analytical methodology. Therefore, microRNAs should not yet be framed as routine clinical biomarkers. Their strongest current relevance lies in multimarker and multi-omics models, where they may help characterize regulatory networks linking environmental exposures, immune signaling, and tissue remodeling [101,102,103].

### 6.5. Environmental Exposures

Environmental exposures are major drivers of epigenetic remodeling in chronic airway disease. Cigarette smoke, particulate matter, air pollution, allergens, respiratory infections, and occupational irritants can all influence DNA methylation, histone modifications, and non-coding RNA expression [28,33,34,104]. These exposures do not act only as external triggers of inflammation; they may also generate persistent molecular changes associated with altered epithelial behavior, immune responsiveness, and potentially disease progression [28,32,34].

Smoking remains the best-established environmental factor linking epigenetic remodeling to COPD. Cigarette smoke induces oxidative stress, epithelial injury, macrophage activation, and persistent inflammatory signaling. These processes are associated with altered DNA methylation and histone-regulatory mechanisms, which may contribute to chronic inflammatory gene expression, impaired repair responses, and corticosteroid resistance. Importantly, smoking-related epigenetic marks may persist beyond active exposure, supporting their relevance as molecular traces of cumulative injury [32,33,34].

Air pollution, particularly fine particulate matter, is increasingly recognized as an epigenetically active exposure relevant to both asthma and COPD. Recent evidence indicates that particulate matter can induce DNA methylation changes, histone modifications, and microRNA dysregulation in pathways related to oxidative stress, inflammation, epithelial dysfunction, and lung injury. These mechanisms provide plausible molecular pathways underlying the association between pollution exposure and worsening respiratory outcomes, especially in vulnerable populations [28].

Allergen exposure may also contribute to epigenetic remodeling, particularly in asthma. Repeated allergen stimulation can influence immune polarization, epithelial cytokine release, type 2 inflammation, and airway hyperresponsiveness. Epigenetic regulation may help explain why allergic inflammation becomes persistent in some individuals despite similar exposure patterns. Within the conceptual framework of this review, epigenetic remodeling may provide a biological link between environmental sensitization, persistent immune regulation, and relatively stable asthma endotypes [29,96,104].

Together, these observations support a model in which environmental exposures interact with genetic susceptibility and innate immune variability to shape chronic airway disease trajectories. Within this model, epigenetic remodeling may be conceptualized as a molecular record of cumulative inflammatory and environmental exposure. This concept is central to the integration of CHI3L1/YKL-40, MBL, and epigenetic signatures into future precision biomarker frameworks for asthma and COPD.

The key biological and clinical characteristics of CHI3L1/YKL-40, MBL, and epigenetic biomarkers are summarized in Table 2.

## 7. The Inflammation–Epigenetic Axis: From Chronic Inflammation to Molecular Memory

Chronic airway diseases are increasingly recognized as disorders of persistent molecular adaptation rather than transient inflammatory activation alone. While inflammatory responses initially serve protective functions, prolonged exposure to cigarette smoke, allergens, air pollution, respiratory infections, and other environmental stimuli may induce biological changes that persist long after the original trigger has diminished. This concept has led to growing interest in the inflammation–epigenetic axis, which describes the transition from dynamic inflammatory signaling to potentially persistent molecular remodeling that may influence long-term disease behavior. Within this framework, chronic inflammation becomes not only a consequence of disease but also may contribute to molecular memory and phenotypic persistence [28,30].

The inflammation–epigenetic axis proposed here represents a broader disease-oriented framework. Trained immunity constitutes one specific mechanism of inflammation-induced molecular memory. It classically refers to persistent functional reprogramming of innate immune cells that alters their response to a subsequent challenge, while related inflammatory-memory phenomena have also been described in epithelial cells [111,112].

As defined in this review, the broader inflammation–epigenetic axis also encompasses persistent transcriptional regulation, oxidative-stress-related epigenetic changes, corticosteroid resistance, and structural airway remodeling that may occur without a formally demonstrated secondary challenge.

### 7.1. NF-κB as a Central Integrator of Inflammatory Signaling

Among the molecular pathways linking inflammation and epigenetic regulation, nuclear factor kappa B (NF-κB) occupies a central position. Activated by cytokines, oxidative stress, microbial products, and tissue injury, NF-κB regulates the transcription of numerous genes involved in immune activation, cytokine production, leukocyte recruitment, and tissue remodeling. Increased or sustained NF-κB signaling has been reported in asthma and COPD and has been implicated in inflammatory amplification, remodeling-associated pathways, and reduced corticosteroid responsiveness. Importantly, NF-κB does not function solely as a transcription factor; it also interacts directly with chromatin-modifying enzymes and epigenetic regulators, thereby linking inflammatory signaling to chromatin regulation and potentially persistent changes in gene expression [34,48,96].

### 7.2. Oxidative Stress as a Driver of Epigenetic Remodeling

Oxidative stress represents a critical intermediary between chronic inflammation and epigenetic modification. Reactive oxygen species generated by activated immune cells, cigarette smoke, environmental pollutants, and damaged epithelial tissues contribute to DNA damage, mitochondrial dysfunction, inflammatory amplification, and altered cellular signaling [6,28,31,32,33].

Consistent with systemic oxidative DNA injury during disease instability, a recent cross-sectional study of patients hospitalized with AECOPD found that serum 8-OHdG concentrations increased across GOLD-defined airflow-limitation stages and were independently associated with both GOLD stage and smoking status [113].

Beyond these immediate effects, oxidative stress influences the activity of DNA methyltransferases, TET enzymes, histone-modifying complexes, and non-coding RNA networks. Consequently, oxidative stress may contribute to the persistence of inflammation-associated epigenetic alterations, thereby promoting long-term changes in airway-cell behavior [6,28,31,32,33].

### 7.3. Epigenetic Remodeling and Stabilization of Inflammatory Programs

Accumulating evidence indicates that repeated inflammatory activation may induce persistent epigenetic alterations that may contribute to the maintenance of disease-associated transcriptional programs. DNA methylation, hydroxymethylation, histone modifications, and microRNA-mediated regulation collectively influence chromatin accessibility and cellular responsiveness to subsequent stimuli. These changes may reinforce pro-inflammatory, profibrotic, or remodeling-associated pathways, and may create a self-reinforcing cycle in which inflammation promotes epigenetic remodeling and altered epigenetic regulation facilitates continued inflammatory signaling. Such mechanisms provide a plausible explanation for the persistence of inflammatory and remodeling-associated airway phenotypes, although their contribution to exacerbations and clinical progression remains insufficiently validated [30,32,33,96].

### 7.4. Trained Immunity and Inflammatory Memory

Recent advances in immunology have introduced the concept of trained immunity, whereby innate immune cells acquire altered responses, while related inflammatory-memory phenomena have been reported in epithelial tissues. Unlike classical adaptive immune memory, trained immunity is mediated largely through metabolic and epigenetic reprogramming. Experimental and translational studies indicate that repeated viral, microbial, or allergic inflammatory stimulation can induce persistent transcriptional or epigenomic changes in airway epithelial and innate immune cells [5,12,13,35]. These findings suggest that chronic airway disease may partially reflect the accumulation of molecular memory encoded through epigenetic remodeling rather than continuous exposure alone [5,12,13,35].

Trained immunity generally requires evidence that a primary exposure induces persistent cellular reprogramming and an altered response to a subsequent challenge. By contrast, many epigenetic alterations described in asthma and COPD have been identified in cross-sectional clinical samples and cannot yet be classified as trained immunity in the strict experimental sense [112].

However, recent airway studies provide direct support for this mechanistic overlap. Repeated rhinovirus exposure can induce persistent DNA-methylation and transcriptional changes in human bronchial epithelial cells and alter their responses to subsequent exposure [12,13]. Epithelial stem cells may retain inflammatory memory after type 2 inflammation. These findings position trained epithelial immunity as one component of the broader inflammation–epigenetic continuum proposed in this review [111].

### 7.5. From Molecular Memory to Phenotypic Stabilization

The biological consequence of inflammation-driven epigenetic remodeling is the stabilization of disease-associated phenotypes. Patients exposed to similar environmental triggers frequently develop markedly different clinical trajectories, suggesting that cumulative molecular adaptation contributes to disease heterogeneity. Epigenetic mechanisms may help explain why some individuals progress toward severe remodeling, recurrent exacerbations, or corticosteroid resistance, whereas others remain relatively stable. Within this conceptual framework, CHI3L1/YKL-40 may be interpreted as a marker of remodeling-associated inflammatory activity, MBL-related measures as indicators of genetically influenced innate immune variability, and epigenetic signatures as candidate markers of cumulative inflammatory and environmental exposure. However, these relationships are not yet established as causal or clinically predictive [10,13,33,34,88,112].

**Figure 3:**
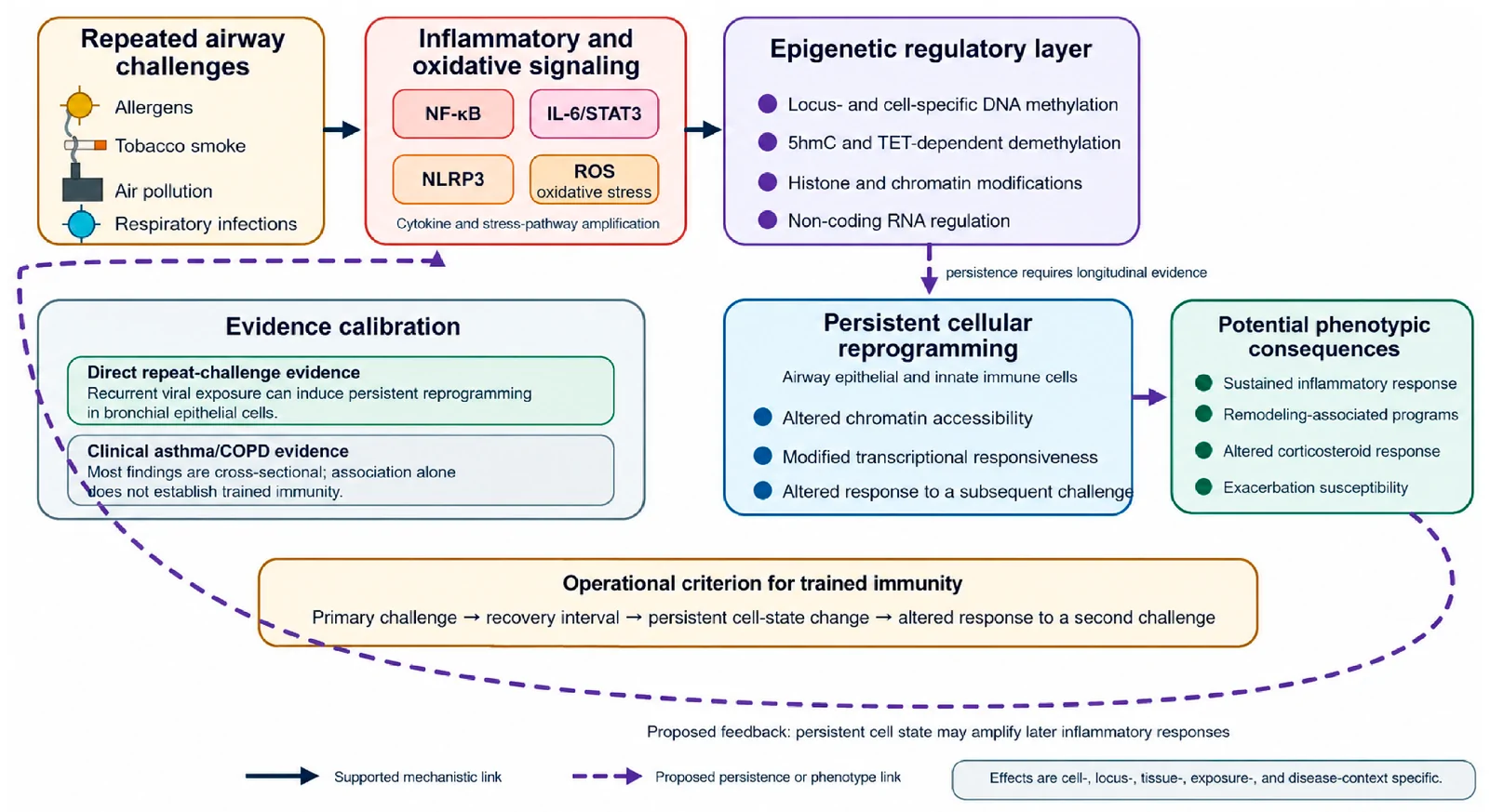
Evidence-calibrated model of the inflammation–epigenetic axis in chronic airway disease. Repeated airway challenges activate inflammatory and oxidative-stress pathways that can induce locus- and cell-specific changes in DNA methylation, hydroxymethylation, chromatin regulation, and non-coding RNA networks [28,31,33,98,101]. Persistent epigenetic reprogramming may alter the responsiveness of airway epithelial and innate immune cells to subsequent challenges. Direct repeat-challenge evidence has been demonstrated in human bronchial epithelial cells following repeated rhinovirus exposure [12], while inflammatory memory has also been identified in human respiratory epithelial progenitor cells following type 2 inflammation [111]. However, most epigenetic findings in asthma and COPD derive from cross-sectional or observational clinical studies and cannot independently establish trained immunity [30,32,95,112]. Potential downstream consequences include sustained inflammatory responsiveness, remodeling-associated transcriptional programs, and altered corticosteroid responsiveness; their effects on exacerbation risk and long-term clinical progression remain insufficiently validated [33,34,96]. Solid arrows indicate relationships supported by direct experimental evidence in the cited cellular or exposure-specific contexts; dashed arrows indicate proposed persistence, clinical consequences, or feedback relationships requiring longitudinal and functional validation. Box colors indicate the main conceptual domains: orange, repeated airway challenges and the operational criterion; red, inflammatory and oxidative signaling; purple, epigenetic regulation; grey, evidence calibration; blue, persistent cellular reprogramming; and green, potential phenotypic consequences.

Together, these biological layers provide a mechanistic framework linking chronic inflammation to long-term disease progression and precision medicine applications in asthma and COPD. Similar multimarker concepts integrating inflammatory and molecular biomarkers have recently been proposed in other chronic disease settings, supporting the transition from single-marker approaches toward systems-based precision medicine [13,33,34,73,112,114,115] (Figure 3).

## 8. Precision Biomarker Models

The growing recognition of biological heterogeneity in asthma and COPD has highlighted important limitations of traditional disease classification systems. Although current clinical frameworks remain indispensable for diagnosis, treatment selection, and disease monitoring, they do not fully capture the molecular heterogeneity underlying individual patient trajectories. Increasing evidence suggests that chronic airway disease should be viewed not only as a physiological disorder characterized by airflow limitation and symptoms, but also as a biologically diverse condition shaped by immune regulation, tissue remodeling, environmental exposure, and epigenetic adaptation. This transition has stimulated the development of precision biomarker models designed to complement conventional clinical assessment with molecular information [86,87,116].

### 8.1. Clinical Phenotyping

Current management strategies for asthma and COPD are largely based on clinical phenotyping frameworks. In COPD, disease severity and treatment decisions are guided primarily by airflow limitation, symptom burden, and exacerbation history as reflected in GOLD recommendations [2]. Similarly, asthma management relies on symptom control, lung function assessment, exacerbation risk, and inflammatory indicators incorporated into GINA guidelines [1]. These approaches have substantially improved standardization of care and remain central to routine clinical practice.

However, patients with similar spirometric parameters frequently exhibit markedly different inflammatory profiles, remodeling activity, therapeutic responsiveness, and long-term outcomes. Forced expiratory volume in one second (FEV1), while clinically valuable, primarily reflects the physiological consequences of disease and provides limited information about the molecular mechanisms responsible for inflammation, remodeling, and progression. Likewise, symptom scores and exacerbation history provide limited insight into the biological processes driving airway inflammation and remodeling [86,87,116].

These limitations have encouraged the development of molecular phenotyping approaches intended to complement conventional clinical classification [86,87,116].

### 8.2. Molecular Phenotyping

The emergence of molecular biomarkers has enabled the transition from descriptive clinical phenotypes toward biologically defined disease endotypes. Within this framework, the greatest potential value of a biomarker lies not only in its association with clinical severity, but also in the complementary biological information it may provide [86,87,116].

CHI3L1/YKL-40 represents one such biomarker. Through its association with macrophage activation, extracellular matrix remodeling, fibrosis, and airway structural adaptation [9,11,68,71,75], CHI3L1/YKL-40 may provide complementary information about remodeling-associated inflammatory activity that is not fully captured by conventional clinical measurements. Elevated CHI3L1/YKL-40 concentrations have been associated with disease severity, airflow limitation, inflammatory burden, and exacerbation-related clinical phenotypes, supporting its investigation as a context-dependent marker of remodeling-associated inflammatory activity [9,11,71].

In contrast, mannose-binding lectin reflects a different dimension of disease biology. MBL2 genotype represents a stable inherited variable, whereas circulating MBL concentrations reflect both MBL2 haplotype and the current infectious or inflammatory context [88]. Differences in complement activation, pathogen recognition, and inflammatory responsiveness may influence host–pathogen interactions and contribute to variation in selected infection-related or inflammatory phenotypes, although the direction and clinical significance of these associations remain inconsistent [25,88,93,94].

Epigenetic signatures provide yet another layer of information. Unlike inherited genetic variants, epigenetic modifications may reflect interactions among environmental exposure, inflammatory signaling, oxidative stress, and tissue adaptation. DNA methylation, hydroxymethylation, histone modifications, and microRNA networks may therefore provide candidate molecular signatures of cumulative exposure and persistent cellular adaptation, and may help characterize the extent to which inflammatory and environmental exposures are associated with persistent molecular adaptation [30,33,34,98].

### 8.3. Multimarker Framework

Because no single biomarker captures the biological complexity of asthma or COPD, future precision-medicine strategies may benefit from integrating complementary molecular and clinical variables [87,116].

These biomarkers were selected because they are hypothesized to provide biologically distinct and potentially complementary information. MBL2 genotype represents inherited variability in lectin-pathway function, whereas circulating MBL concentrations also depend on the infectious and inflammatory context [88,93,94]. CHI3L1/YKL-40 reflects inflammation-associated remodeling and extracellular-matrix turnover [9,11,68,71,75]. Epigenetic signatures may reflect cumulative exposure and persistent cell-intrinsic adaptation [13,30,33,34]. Their proposed complementarity is therefore informational rather than diagnostic: each layer may contribute a distinct variable to a disease-specific model, but none is expected to classify asthma or COPD in isolation.

Mechanistically, these biomarker layers are best viewed as components of a context-dependent network rather than as molecules with a demonstrated direct physical interaction. MBL2 genotype strongly influences circulating MBL concentrations and lectin-pathway activity, but the airway consequences appear to depend on the infectious and inflammatory context: differences in microbial clearance and host–microbe interactions may modify infectious burden and the resulting cytokine and oxidative-stress environment [26,88,93,94,117].

Recent exploratory findings in patients hospitalized with AECOPD further indicate that relationships among circulating inflammatory biomarkers may vary across GOLD-defined airflow-limitation stages, illustrating that biomarker interpretation may depend on disease severity and network context [118].

Host–microbe and inflammatory signals can activate NF-κB-dependent inflammatory pathways. Separately, CHI3L1/YKL-40 expression is associated with inflammatory and tissue-injury contexts, but a direct MBL–NF-κB–CHI3L1 pathway has not been demonstrated in asthma or COPD [10,48,75].

Experimental studies independently implicate CHI3L1/YKL-40 in macrophage activation, fibroblast responses, and remodeling-associated extracellular-matrix regulation [68,69,71,74,75]. In parallel, repeated inflammatory and oxidative exposures can alter DNA methylation, hydroxymethylation, histone-regulatory mechanisms, and non-coding RNA networks [28,30,32,33,34,95,96,97,98,99]. Persistent epigenetic reprogramming may, in turn, modify the magnitude and duration of subsequent inflammatory and remodeling responses, thereby generating cell-intrinsic inflammatory memory after repeated exposure [5,12,13,48,111,112].

The proposed framework integrates an upstream host-defense modifier, a remodeling-associated response marker, and candidate molecular signatures of cumulative cellular adaptation within a testable host–microbe–inflammation–remodeling axis.

Age and developmental context require separate consideration. Evidence for CHI3L1/YKL-40 and MBL in asthma includes pediatric cohorts [20,22,72,82], whereas COPD studies predominantly concern adults with longer cumulative smoking and environmental exposure [11,68,71,79]. Immune maturation, exposure history, treatment, comorbidity, and age-related epigenetic variation may alter both baseline biomarker distributions and biomarker–outcome associations [28,30,31,32]. Thresholds and multimarker models should therefore not be transferred uncritically between pediatric asthma, adult asthma, and COPD; these populations require separate reference distributions and external validation.

This framework does not imply identical biomarker performance across asthma and COPD. The relative contribution and clinical interpretation of each biomarker are expected to vary according to disease-specific inflammatory endotypes. For example, CHI3L1/YKL-40 may reflect a combination of inflammatory and remodeling-associated activity in both diseases [10,11,19], whereas its association with eosinophilic or neutrophilic inflammation may differ between asthma phenotypes and COPD. Likewise, the clinical significance of MBL and epigenetic signatures is likely influenced by disease-specific immune mechanisms and environmental exposures [25,29,33,88]. Future multimarker algorithms should be developed and validated in an endotype-specific rather than universal manner.

Advances in machine learning, systems biology, and multi-omics integration are increasingly facilitating the development of predictive models capable of combining clinical variables with molecular information. Such approaches may support the development of models for disease stratification and outcome prediction, but their incremental value over established clinical variables remains to be demonstrated through external validation [119,120,121,122,123]. Although substantial validation is still required before routine implementation, multimarker frameworks represent a promising step toward biologically informed respiratory medicine [119,122,123].

**Figure 4:**
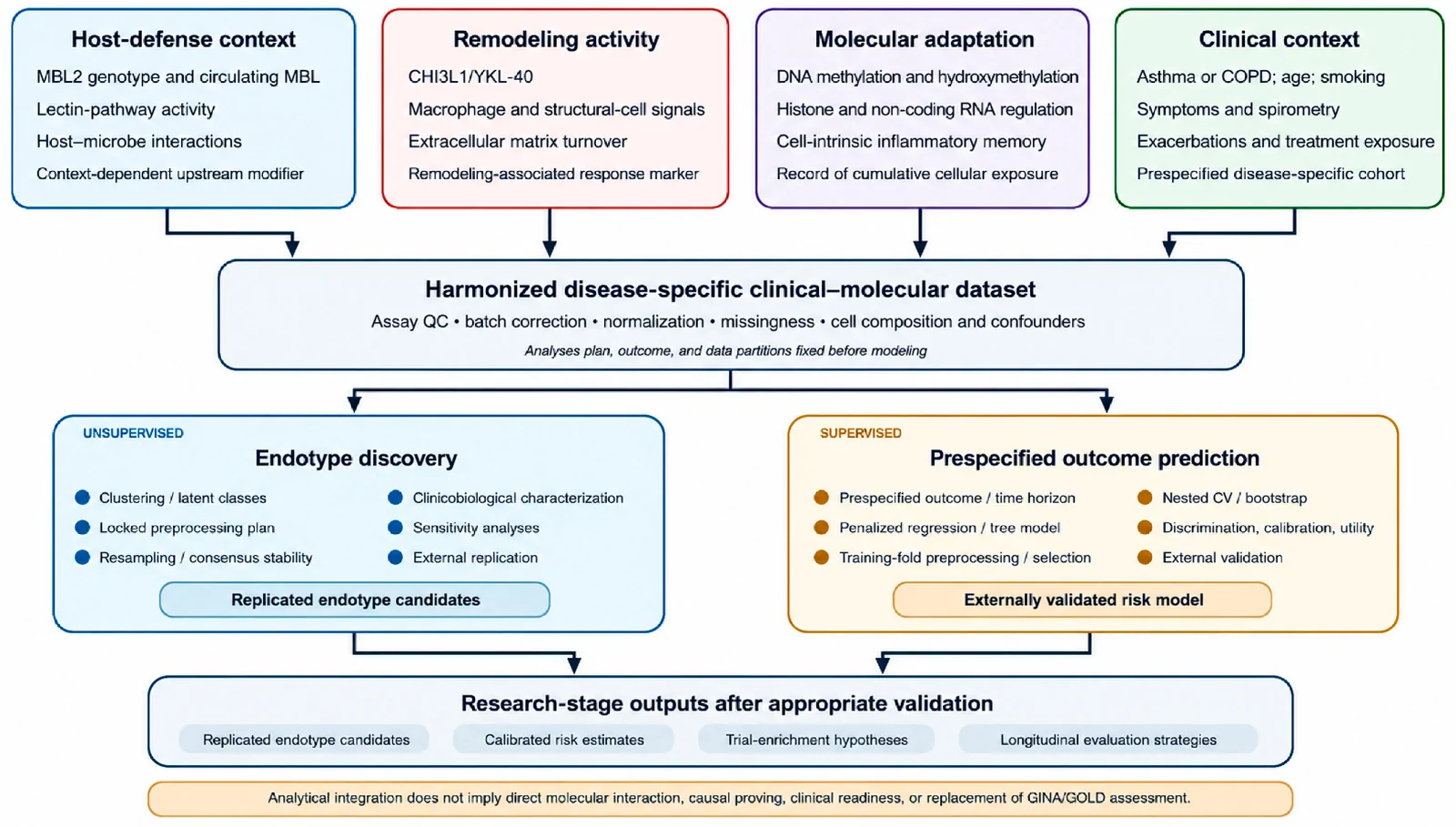
Conceptual multimarker–AI workflow for asthma and COPD. MBL2 genotype and circulating MBL represent context-dependent host-defense variability [26,88,93,94]; CHI3L1/YKL-40 is treated as a marker of remodeling-associated activity [10,68,71,72,75]; and epigenetic signatures capture cumulative inflammatory exposure [30,33,34] and cell-intrinsic memory [12,13]. These layers are combined with prespecified clinical variables in a disease-specific dataset. Unsupervised analyses use clustering or latent-class methods followed by resampling-based stability assessment [57], clinical and biological characterization, sensitivity analyses, and external replication. Supervised prediction prespecifies the outcome and time horizon; preprocessing and feature selection are performed within training folds, followed by nested cross-validation or bootstrapping, evaluation of discrimination, calibration and utility, and external validation [57,119,120,121,125,126,127,128]. Outputs remain research-stage candidates until independently validated. Arrows indicate analytical integration rather than direct molecular interaction, causal synergy, clinical readiness, or replacement of GINA/GOLD assessment. Box colors indicate the functional domains of the workflow: light blue, host-defense context; light red, remodeling activity; light purple, molecular adaptation; light green, clinical context; pale blue-grey, the harmonized disease-specific clinical–molecular dataset and research-stage outputs; blue, unsupervised endotype discovery; orange, supervised outcome prediction and validation-related elements; pale grey, research outputs after validation, and pale orange, methodological cautions and interpretive limitations.

Rather than replacing established clinical frameworks such as GOLD and GINA, future biomarker-driven models will likely function as complementary tools capable of refining disease classification and improving individualized care [1,2]. Figure 4 illustrates a staged analytical workflow in which complementary biological layers are combined with disease-specific clinical variables for endotype discovery and outcome prediction, followed by explicit model validation [119,120,121,124,125,126].

## 9. Translational Perspectives

The growing understanding of chronic airway disease biology is creating new opportunities for the translation of molecular discoveries into clinical practice. Traditional disease assessment relies predominantly on physiological measurements, symptom burden, and exacerbation history. Several of the biomarkers emphasized in this review, particularly circulating CHI3L1/YKL-40 and MBL, can be measured in serum or plasma, whereas epigenetic remodeling and inflammatory-memory processes are strongly dependent on the sampled tissue and cell population. Consequently, circulating biomarker measurements should be interpreted as potential indirect surrogates whose relationship to localized airway biology requires explicit validation [129].

Future studies integrating blood-based biomarkers with bronchial biopsy specimens, bronchoalveolar lavage, induced sputum, single-cell transcriptomics, spatial transcriptomics, and epigenomic profiling are needed to determine how accurately circulating biomarkers reflect local airway molecular memory.

Clinical translation should begin with a prespecified context of use rather than with a universal biomarker score. CHI3L1/YKL-40 could be evaluated for characterizing remodeling-associated activity or exacerbation-associated inflammatory phenotypes [11,18,71], MBL as a context-dependent modifier of infection-related phenotypes, and epigenetic signatures as indicators of cumulative exposure or corticosteroid-response pathways [18,33,34,42,71,93,94,96].

These measurements should complement, rather than replace, established GINA/GOLD-based assessment [86,87,116].

Trial enrichment and longitudinal response monitoring represent potential contexts of use that require prospective evaluation. They should not be regarded as validated therapeutic targets. Before clinical implementation, each biomarker panel requires analytical standardization, demonstration of incremental value beyond symptoms, spirometry, and exacerbation history, external validation, and prospective evidence that biomarker-guided decisions improve patient outcomes [86,116,125,126]. At present, the proposed framework should be considered a research model rather than a diagnostic algorithm.

### 9.1. Biomarker Panels Rather than Single Biomarkers

Multimarker panels should be evaluated against a prespecified clinical baseline rather than against single biomarkers alone. The relevant question is whether CHI3L1/YKL-40, MBL, and epigenetic variables provide reproducible improvement in discrimination, calibration, or clinical utility beyond age, smoking exposure, spirometry, symptoms, and exacerbation history. Because their distributions and biological meaning differ by disease and endotype, asthma and COPD should be modeled separately or using prespecified interaction terms, followed by external validation [100,104,119,125,130,131].

### 9.2. Artificial Intelligence and Machine Learning

The increasing availability of high-dimensional biological data has created significant opportunities for artificial intelligence (AI) and machine learning applications in respiratory medicine. Modern computational approaches can integrate clinical variables, pulmonary function measurements, imaging findings, laboratory biomarkers, genomic information, and epigenetic profiles into unified predictive models. Such strategies may reveal biologically meaningful disease subgroups that remain undetectable through conventional clinical assessment alone [119,120,121,127].

A useful computational strategy would distinguish endotype discovery from outcome prediction. After harmonizing clinical and molecular variables, unsupervised hierarchical clustering or latent-class analysis could identify patient groups defined by CHI3L1/YKL-40, MBL, epigenetic signatures, and conventional clinical features within disease-specific cohorts, without imposing predefined endotype labels [57,119,120,121].

These clusters would require biological interpretation and replication in independent cohorts. For predefined outcomes, such as exacerbations, lung-function decline, or treatment response, penalized regression, random forests, or gradient-boosting models could test whether molecular data improve prediction beyond age, smoking, spirometry, and previous exacerbations [125,126,128]. Because these datasets frequently contain many variables but relatively few patients, all preprocessing, imputation, feature selection, and model tuning should be restricted to the training data within an appropriate resampling framework, such as nested cross-validation [125,126,128]. Feature-attribution methods may indicate which biomarker layer contributes to a prediction, but they do not establish causality. AI should therefore be used as a framework for hypothesis testing and model validation rather than presented as evidence of a clinically ready algorithm.

### 9.3. Multi-Omics and Precision Medicine

The future of precision respiratory medicine will likely depend on the integration of multiple molecular data sources rather than reliance on individual biomarkers. Advances in genomics, transcriptomics, proteomics, metabolomics, microbiomics, and epigenomics are progressively improving our understanding of disease heterogeneity and biological endotypes. When combined with clinical and physiological information, these datasets may facilitate the development of individualized disease models that can be evaluated for prediction of progression, exacerbations, and treatment response [87,119,123].

Within this evolving framework, CHI3L1/YKL-40, MBL, and epigenetic markers may represent candidate translational biomarker layers with differing levels of accessibility and clinical maturity [13,30,34,88,93].

### 9.4. Future Directions

Future translation requires a staged evidence pathway: analytical assay validation, prospective evaluation in disease- and endotype-specific longitudinal cohorts, external validation of multimarker models, and ultimately interventional trials testing whether biomarker-guided decisions improve patient outcomes beyond guideline-based care [1,2,86,87,116,125,126].

## 10. Limitations

Several limitations should be considered when interpreting the concepts discussed in this review.

First, the available evidence regarding CHI3L1/YKL-40 [19,84], mannose-binding lectin (MBL) [91,93,94], and epigenetic biomarkers [29,32,34] remains heterogeneous with respect to study design, patient populations, disease definitions, analytical methodologies, and clinical endpoints. Such variability complicates direct comparison across studies and may limit the generalizability of individual findings [34,86,87].

Associations between circulating CHI3L1/YKL-40 and clinical outcomes do not establish that the circulating protein directly drives disease progression. MBL2 genotype influences MBL production and lectin-pathway activity, but its downstream effects on asthma and COPD outcomes remain context-dependent and inconsistent. Likewise, epigenetic alterations may function as mediators, consequences, or correlates of chronic inflammation, and cross-sectional associations cannot determine their causal direction [10,30,32,33,79,88,93,94].

Epigenetic remodeling represents a highly dynamic and context-dependent process influenced by age, environmental exposure, medication use, smoking status, comorbidities, disease duration, sampled tissue and cell composition. Consequently, epigenetic signatures may not transfer across tissues, cell populations, exposure histories, or clinical populations without explicit external validation [28,32,33].

Furthermore, while the concept of inflammation-driven molecular memory provides an attractive framework for understanding disease persistence and heterogeneity, the current evidence base remains incomplete. Many aspects of trained immunity, epigenetic adaptation, and long-term airway remodeling are still under active investigation, and their clinical implications have not yet been fully established [5,12,13].

Finally, the multimarker framework proposed in this review should be regarded as a conceptual and translational model rather than a validated clinical algorithm. Although advances in systems biology, multi-omics integration, and artificial intelligence have created new opportunities for precision respiratory medicine, prospective validation studies are required before such approaches can be routinely implemented in clinical practice [86,87,123,125,126].

## 11. Conclusions

CHI3L1/YKL-40, MBL-related measures, and epigenetic signatures may provide distinct but non-specific information on remodeling-associated inflammatory activity, context-dependent host defense, and persistent cellular adaptation in asthma and COPD. Their integration is conceptually plausible, but neither direct molecular synergy nor combined clinical utility has been established.

Future models should be developed separately in disease- and endotype-specific cohorts using standardized assays and matched blood and airway samples. Their incremental value beyond symptoms, spirometry, smoking exposure, and exacerbation history should be assessed for prespecified outcomes and time horizons. Adequate discrimination, calibration, clinical utility, and external validation must be demonstrated before biomarker-guided trials are undertaken. Until these requirements are met, the proposed multimarker framework should remain a hypothesis-generating research strategy rather than a diagnostic or therapeutic algorithm.

## Figures and Tables

**Table 1:** Epigenetic mechanisms and current translational status in asthma and COPD.

Epigenetic Mechanism	Principal Mechanism	Relevance in Asthma/COPD	Current Translational Status
DNA methylation [30,32,95,96]	DNMT-mediated 5mC formation can alter transcriptional activity	Asthma endotypes and severity, COPD airflow limitation	Candidate molecular-phenotyping marker; not clinically validated
DNA hydroxymethylation [29,31,33,97,98]	TET-mediated 5mC oxidation and active demethylation	Airway epithelial adaptation, oxidative stress response, experimental allergic-airway regulation; phenotype-level evidence remains limited	Discovery-stage biomarker layer
Histone modifications [29,33,34,96,97,99,100]	H3K27 acetylation and HDAC2-dependent transcriptional repression	Epithelial inflammatory activation in asthma; impaired corticosteroid responses in COPD	Mechanistic endotyping and therapeutic targeting; not a routine biomarker
miRNA [101,102,103]	Post-transcriptional repression of target mRNAs	Oxidative stress, apoptosis, inflammation, and remodeling networks	Candidate panel component requiring prospective validation
Environmental epigenetic imprinting [28,32,97,104]	Persistent regulatory changes following inhaled environmental exposures	Smoking-, pollution-, and allergen-associated molecular memory	Exposure-associated biomarker discovery, risk stratification

Legend: DNA—deoxyribonucleic acid; DNMT—DNA methyltransferase; 5mC—5-methylcytosine; H3K27—histone H3 on lysine 27; mRNA—messenger ribonucleic acid; COPD—chronic obstructive pulmonary disease; TET—ten-eleven translocation enzyme; HDAC2—histone deacetylase 2.

**Table 2:** Complementary information provided by CHI3L1/YKL-40, MBL, and epigenetic biomarker layers in asthma and COPD.

Feature	CHI3L1/YKL-40	MBL	Epigenetic Markers
Representative assay	Serum or plasma CHI3L1/YKL-40 immunoassay [9,18]	Serum or plasma MBL measurement; MBL2 genotyping when relevant [88]	DNA methylation, 5hmC, histone, or miRNA profiling in blood or airway-derived cells [30,98,101]
Biological information	Remodeling-associated inflammatory and tissue-injury activity [10,68,71]	Genetically influenced lectin-pathway and host-defense capacity [25,88]	Cell-state adaptation and exposure-associated gene regulation [28,33,104]
Major determinants and confounders	Inflammation, tissue injury, age, and comorbidity and assay-dependent thresholds [11,71,82]	MBL2 haplotype and the infectious or inflammatory context [88,93,94]	Cell composition, tissue source, environmental exposure, age, and treatment [32,33]
Potential context of use	Characterisation of remodeling-associated or exacerbation-associated risk [11,71,82]	Infection-related risk stratification in selected settings [93,94]	Exposure-related or inflammatory-memory endotyping [29,30,32]
Key limitation	Limited disease specificity and non-standardized thresholds [10,18,84]	Context-dependent and inconsistent clinical associations [91,93,94]	Strong tissue and cell specificity; absence of a standardized panel [29,32,33]
Current clinical status	Investigational adjunct; not diagnostic [10,18]	Investigational context modifier; not diagnostic [93,94]	Discovery-stage biomarkers; not clinically validated [29,32,33]

Legend: CHI3L1/YKL-40—chitinase-3-like protein 1; MBL—mannose-binding lectin; MBL2—mannose-binding lectin 2 gene; DNA—deoxyribonucleic acid; 5hmC—5-hydroxymethylcytosine; miRNA—microRNA. These layers provide complementary but non-interchangeable information. Their comparison does not imply direct molecular interaction, causal synergy, or validated clinical utility.

## Data Availability

No new datasets were generated or analyzed during the preparation of this review. All information discussed in this manuscript was obtained from previously published studies cited in the reference list. Further information supporting the findings and interpretations presented in this review is available from the corresponding author upon reasonable request.

## Abbreviations

The following abbreviations are used in this manuscript:

Abbreviation	Definition
5hmC	5-Hydroxymethylcytosine
5mC	5-methylcytosine
8-OHdG	8-Hydroxy-2′-Deoxyguanosine
ACO	Asthma–COPD Overlap
AECOPD	Acute Exacerbation of Chronic Obstructive Pulmonary Disease
AI	Artificial Intelligence
AKT	Protein Kinase B
CHI3L1/YKL-40	Chitinase-3-Like Protein 1
COPD	Chronic Obstructive Pulmonary Disease
CpG	Cytosine–phosphate–guanine dinucleotide
DNA	Deoxyribonucleic Acid
DNMT	DNA Methyltransferase
ECM	Extracellular Matrix
ERK	Extracellular Signal-Regulated Kinase
FEV1	Forced Expiratory Volume in One Second
FVC	Forced Vital Capacity
GINA	Global Initiative for Asthma
GOLD	Global Initiative for Chronic Obstructive Lung Disease
H3K27ac	Histone H3 Lysine 27 Acetylation
HDAC	Histone Deacetylase
HDAC2	Histone Deacetylase 2
IL	Interleukin
IL-13Rα2	Interleukin-13 Receptor Alpha 2
lncRNA	Long Non-Coding RNA
MAPK	Mitogen-Activated Protein Kinase
MBL	Mannose-Binding Lectin
MBL2	Mannose-Binding Lectin 2 Gene
miRNA	MicroRNA
ncRNA	Non-Coding RNA
NF-κB	Nuclear Factor Kappa B
NLRP3	NOD-, LRR-, and Pyrin Domain-Containing Protein 3
OMIC	High-throughput molecular profiling technologies (e.g., genomics, transcriptomics, proteomics, metabolomics, epigenomics, and microbiomics)
PAR-2	Protease-Activated Receptor 2
RNA	Ribonucleic Acid
ROS	Reactive Oxygen Species
STAT3	Signal Transducer and Activator of Transcription 3
T2	Type 2 Inflammation
TET	Ten-Eleven Translocation
TET1	Ten-Eleven Translocation 1
Th	T Helper Cell
TMEM219	Transmembrane Protein 219
TNF-α	Tumor Necrosis Factor Alpha
YKL-40	Chitinase-3-Like Protein 1 (CHI3L1)
